# The influence of cardiac substructure dose on survival in a large lung cancer stereotactic radiotherapy cohort using a robust personalized contour analysis

**DOI:** 10.1016/j.phro.2024.100686

**Published:** 2024-12-01

**Authors:** Luuk H.G. van der Pol, Jacquelien Pomp, Firdaus A.A. Mohamed Hoesein, Bas W. Raaymakers, Joost J.C. Verhoeff, Martin F. Fast

**Affiliations:** a Department of Radiotherapy, University Medical Center Utrecht, Heidelberglaan 100, 3584 CX Utrecht, the Netherlands; b Department of Radiology, University Medical Center Utrecht, Heidelberglaan 100, 3584 CX Utrecht, the Netherlands; c Department of Radiation Oncology, Amsterdam UMC Location University of Amsterdam, Amsterdam, the Netherlands

**Keywords:** Cardiac substructures, Lung cancer, Overall survival, Contour variability, Modelling variability, Deep learning

## Abstract

Background/Purpose: Radiation-induced cardiac toxicity in lung cancer patients has received increased attention since RTOG 0617. However, large cohort studies with accurate cardiac substructure (CS) contours are lacking, limiting our understanding of the potential influence of individual CSs. Here, we analyse the correlation between CS dose and overall survival (OS) while accounting for deep learning (DL) contouring uncertainty, α/β uncertainty and different modelling approaches. Materials/Methods: This single institution, retrospective cohort study includes 730 patients (early-stage tumours (I or II). All treated: 2009–2019), who received stereotactic body radiotherapy (≥ 5 Gy per fraction). A DL model was trained on 70 manually contoured patients to create 12 cardio-vascular structures. Structures with median dice score above 0.8 and mean surface distance (MSD) <2 mm during testing, were further analysed. Patientspecific CS dose was used to find the correlation between CS dose and OS with elastic net and random survival forest models (with and without confounding clinical factors). The influence of delineation-induced dose uncertainty on OS was investigated by expanding/contracting the DL-created contours using the MSD ± 2 standard deviations. Results: Eight CS contours met the required performance level. The left atrium (LA) mean dose was significant for OS and an LA mean dose of 3.3 Gy (in EQD2) was found as a significant dose stratum. Conclusion: Explicitly accounting for input parameter uncertainty in lung cancer survival modelling was crucial in robustly identifying critical CS dose parameters. Using this robust methodology, LA mean dose was revealed as the most influential CS dose parameter.

## Highlights

• Cardiac auto-contouring using deep learning in a large lung radiotherapy cohort.
• Novel method to account for delineation-induced dose uncertainty in survival models.
• Taking into account α/β uncertainty in the modelling.
• Elastic net and random survival forest models to resolve dose parameter correlation.
• Left atrium mean dose significant for overall survival.

## 1 Introduction

Since the Radiation Therapy Oncology Group (RTOG) 0617 [1] study found lower survival in lung cancer patients in the treatment arm with a higher dose, and later correlated higher heart dose to lower overall survival (OS), dose to the heart has been increasingly investigated [2]. This has led to several studies linking dose to the heart and lower OS [3], [4]. However, the exact causal link is not well understood. Earlier studies focus on the whole heart (WH) as one organ at risk, but more recent studies investigate cardiac substructures (CS). Dose to several CS is suspected to negatively affect OS [5], [6], [7], [8], [9], [10], [11], [12], [13], [14], [15], [16], [17]. However, as CS contours are not routinely available in clinics, most studies have small cohorts [5], [6], [7], [8], [9], [10], [11], [12], [13], [14], [15], making the results less robust. Studies investigating larger cohorts mostly rely upon deformable image registration (DIR), as manual contouring is too time-consuming to perform retrospectively for a large cohort. In these studies, DIR is used to deform the anatomy of a template patient, for whom CS contours are available, to the anatomy of patients without CS contours. Although DIR can deform similar anatomies, it struggles when anatomical differences become larger [18]. This might explain why contouring results from studies by McWilliam [16] and Stam [17], using DIR were outperformed by studies with manual delineations and strict delineation guidelines, e.g. Zhou (showing lower centre-of-mass deviation compared to McWilliam et al.) [19] and Stockinger (showing higher Dice score and lower mean surface distance compared to Stam et al.) [20]. In contrast to DIR, other automatic segmentation methods reach manual delineation performance [21], [22], [23].

This study aims to determine the most influential CS concerning OS in lung cancer patients treated with stereotactic body radiotherapy (SBRT). To this end, individual CS contours are generated using deep learning (DL), thereby circumventing the registration inaccuracies that persist in DIR. These CS contours are then used to extract CS dose parameters for survival modelling. The influence of contour uncertainty on the survival modelling is also investigated, first, by expanding, and second, by contracting the DL-created contours.

Additionally, the effect of confounding factors and different α/β values on survival modelling outcomes is investigated. Finally, we identify CS dose levels that are predictive of OS.

## 2 Materials and methods

### 2.1 Patient characteristics

Included were 892 lung cancer patients treated with SBRT at a single centre between 2009 and 2019, for whom CT scans, treatment plans and clinical information were available. All included patients received SBRT (*≥* 5 Gy). After exclusion for re-irradiation, palliative treatment or data usage objection, 820 patients remained. Therefrom the 730 patients with early tumour stages (I or II) were selected for further analysis.

The following patient characteristics were retrieved for these patients (either from electronic reports or from imaging data): sex, age, tumour stage, laterality, planning target volume (PTV), survival time, pathology, and distance between treatment target and the heart. This distance was defined for PTV and GTV separately, both their 3D distances were split into cranio-caudal (CC) and in-plane distances. Survival time was defined to start after the final day of treatment. Detailed patient characteristics are shown in Table 1.

**Table 1:** Patient characteristics and completeness of data for the cohort in this study.

**Category**	**N/median (range or percentage of total)**
Sex	
Male	412 (56.4%)
Female	318 (43.6%)
Age	74 (37–92)
Tumour stage	
1	559 (76.6%)
2	171 (23.4%)
Laterality	
Left	314 (43.0%)
Right	416 (57.0%)
Gross tumour volume (GTV)	9.8cc (0.4–389.1) 100% complete
Planning target volume (PTV)	20.4cc (1.9–502.2) 100% complete
Survival/censoring time	1267 days (11–4737) 100% complete
Distance to heart (PTV) (CC)	24 mm (−42–123) 100% complete
Distance to heart (PTV) (in-plane)	31 mm (0–105) 100% complete
Distance to heart (GTV) (CC)	27 mm (−42–126) 100% complete
Distance to heart (GTV) (in-plane)	34 mm (0–105) 100% complete
Pathology	
Squamous	63 (8.6%)
Adeno	88 (12.1%)
Proven	538 (73.7%)
Unknown	41 (5.6%)
Total	730 (100%)
Fractionation schedules	
3x18 Gy	176 (24.1%)
5x12 Gy	368 (50.4%)
8x7.5 Gy	140 (19.2%)
12x5 Gy	41 (5.6%)
Others	5 (0.7%)

**Fig. 1:**
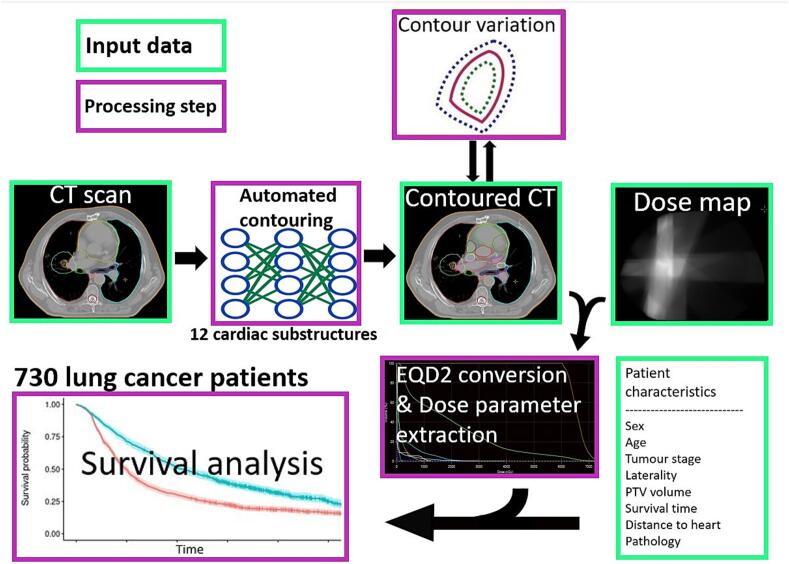
Overview of the entire method.

Fig. 1 shows a detailed overview of the methodology used in this study.

This study did not fall under the scope of the Dutch Medical Research Involving Human Subjects Act (WMO) due to its retrospective nature. Therefore, it did not require approval from an accredited medical ethics committee in the Netherlands. However, in our centre, an independent quality check was conducted to ensure compliance with legislation and regulations (regarding the Informed Consent procedure, data management, privacy aspects and legal aspects).

### 2.2 Contour creation and evaluation

Planning CT scans of 70 lung cancer patients were randomly selected and used for the training of the DL model. This data was split between training, validation, and testing, with 50, 10, and 10 patients respectively. Input CT scans for the DL model were either motion-averaged or time-weighted motion averaged (known as mid-position), non-contrast CT scans, created from 4D-CTs. Following visual assessment, the CT scans were deemed of comparable quality throughout the cohort.

To limit the risk of underperformance of the model due to missed systematic differences/changes, CT scans for training were selected throughout the whole inclusion period.

Clinically available structures of interest that were present for every patient in this cohort are the left lung (LL), right lung (RL), and the whole heart (WH). CS and cardio-pulmonary structures were manually contoured by a junior researcher (LP) on the 70 selected scans and revised or approved by an experienced cardiothoracic radiologist (FM). The following structures were contoured: left atrium (LA), left ventricle (LV), right atrium (RA), right ventricle (RV), aortic valve (AV), ascending aorta (AA), superior vena cava (SVC), inferior vena cava (IVC), pulmonary artery (PA), pulmonary veins (PV), and the coronary sinus (CorSin). Contouring of the four chambers was done following the guidelines from Feng et al. [24]. Here, the AA was defined as the part of the aorta that is still within the heart structure. The PA was contoured from the PA root as far as the second bifurcation. Both the SVC and IVC were contoured from where separation from the RA can be identified, the SVC as far as the height of the aortic trunk and the IVC until it disappears in the liver. The CorSin was contoured between the RA and LA but cannot always be seen. The PVs were contoured from the LA until their first bifurcation. The coronary arteries were not included in this study as they are difficult to outline reproducibly on non-gated, non-contrast planning CT scans and thereby are unlike to meet the criteria set below [25].

The DL model that was used is a nnU-Net model [26], which outperformed other DL models during initial testing. The network was trained on whole-volume CT scans with a typical size of 512×512×117 pixels and typically 1x1x3 mm resolution (with 0.10, 0.10, and 0.02 mm standard deviation (SD) respectively). The model was trained using 5-fold cross-validation, with 1000 epochs for each fold using the provided default settings. Performance on the test set was evaluated based on dice score (DSC), 95% Hausdorff distance (HD) and MSD. The performance will be shown as median (range) for DSC and 95% HD, where mean (SD) will be shown for mean surface distance (MSD). SD is shown for MSD, as this will be used to capture the 95% confidence interval of contours elaborated in the next paragraph. Only structures with median DSC above 0.80 and MSD < 2 mm were deemed sufficiently accurate to be used in the further analysis, following the guidelines of AAPM taskgroup 132 [27].

### 2.3 Robust dose metric analysis

The MSD and SD that resulted from the auto-contour test set performance were used as a margin to create expanded (MSD + 2 SD) and contracted (MSD − 2 SD) variations for each created contour. This method was used to estimate delineation-induced dose evaluation uncertainty. Dose-survival relationships derived from the original contours are required to stay significant with the contour variations, which simulate systematic under/over-segmentation, to be considered robust findings.

Dose-volume histogram (DVH) points were extracted based on the planned dose per contour of interest, for all contour variations, using an in-house developed script for MATLAB (version 2019b, Natick, Massachusetts: The MathWorks Inc.). The dose was converted to 2 Gy equivalent fraction dose (EQD2), using an α/β of 2 Gy for the WH and CS, and 3 Gy for the other structures. Furthermore, robustness was also tested by using an α/β of 3 Gy for the WH and CS. Thereafter, Dmean and D0.1 cc were extracted, where D0.1 cc was used as a surrogate for Dmax. The α/β ratios of 2 and 3 Gy for the WH and CS were used before by others [16], [17], and 3 Gy for the other structures is currently used in our clinical practice. Dose constraints for conventional organs at risk can be found in Supplementary Materials 1 Table 1.

### 2.4 Survival modelling & statistical analysis

The survival modelling and statistical analysis were performed using Rstudio (Version 4.0.4 [28]). First, univariable Cox-regression analysis (UVA) was used to find which patient characteristics were significant confounding factors. Multivariable Cox-regression fails to resolve collinearity between CS dose parameters adequately, thus models with shrinkage techniques were investigated. Ridge [29] and Lasso [30] regression were initially tested but over-fitted and under-fitted the data respectively. An elastic net (EN) [31] approach was taken to resolve these problems, wherein an optimization parameter alpha is tuned between 0 (Ridge regression) and 1 (Lasso regression). Single runs can be unstable as a random data partition is selected in each iteration, therefore 1000 bootstraps were performed. More information about the use of EN models in this study is outlined in Supplementary Materials 2. The selection of dose parameters in the EN models was evaluated as a percentage of the total number of bootstraps. Dose parameters that are selected more often are more important for an accurate survival model, thus play a more prominent role in patient survival.

Besides EN, random survival forests (RSFs) models were used for the same goal of determining the most influential dose parameters [32]. Next, 500 RSFs were created per model variation. Each RSF can identify the most important parameters in a set of correlated data [33]. Each forest was allowed 500 trees. Implementation and standard settings are available online [34]. Additional information explaining how the importance of a parameter was established is outlined in Supplementary Materials 2. Dose parameters with a median importance value above 0.01 out of the RSFs were considered the most important [33].

Each approach (EN or RSF) featured two times four different model runs: three for the contour variations (contracted/original/expanded) and one using an α/β of 3 Gy for all structures (with original contours). All four of these models were run twice, once using only dose parameters as input (dose-only models) and once using the dose parameters plus confounding factors as input (complete models).

Dose parameters that were considered to be the most influential in both the RSFs and EN models, along with the confounding factors, were used to build a final multivariable Cox-regression model.

### 2.5 Dose threshold determination

Optimal splits were determined for each of the most influential dose parameters to define potential dose thresholds. This was done by randomly selecting two-thirds of the data per run and repeating the process 100 times, as single data partitions might not reflect the entire dataset. The median value was taken from these 100 runs and used as a stratum for a Kaplan-Meier plot. The median survival times were then extracted to inspect the potential gain in survival. The process was repeated using all contour variations and the different α/β value to ensure robustness.

## 3 Results

**Fig. 2:**
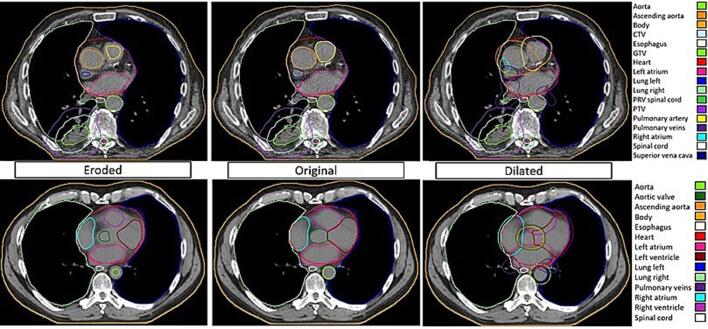
Deep learning outcomes and contour variations for two example patients. Window/level settings are 400/40 Hounsfield units.

Training the auto-contouring model took 130 hours using 8 GB of RAM. DL contour creation took two minutes per patient. The contours created by the trained nnU-Net on the test patient’s CT scans were evaluated against manual ground truth contours. This led to the inclusion of the LA, RA, RV, LV, aorta, SVC, IVC and PA for further analyses while excluding the smaller structures for which contour atlases were not applied (AA, AV, CorSin, and PV). Performance scores for the test set can be seen in Supplementary Materials 3 Table 3. Additionally, 50 patients were visually inspected at random, for which no large mistakes were detected. The outcomes of the automated contouring process of two example patients are shown in Fig. 2.

UVA showed significant impact on survival for age, sex, PTV, and pathology, as well as dose to each CS, the whole heart and individual lungs. Tumour laterality, tumour stage, laterality, and distance between the target (both GTV and PTV, both CC and in-plane distance) and the heart were not significantly associated with survival and therefore not used in the further analysis. UVA results for these confounding factors are shown in Supplementary Materials 4 Table 4.

For the EN modelling approach, the mean tuned alpha parameters per model variation were: 0.01 (original, dose only), 0.00 (expanded, dose only), 0.01 (contracted, dose only), 0.03 (α/β of 3 Gy, dose only), 0.10 (original, complete), 0.05 (expanded, complete), 0.10 (contracted, complete), and 0.12 (α/β of 3 Gy, complete). This revealed more ridge-like models (alpha of 0) when only dose is included, which led to more frequent selection of dose parameters (except in the case of the adjusted α/β, as can be seen in Table 2.) The dose-only EN results pointed towards LA mean dose as the most important parameter, with an average selection percentage of 78 % over the different model configurations (Table 2).

**Table 2:** Combined results from the survival modelling approaches. RSF values are only noted if above the importance threshold (0.01). Abbreviations: LA = left atrium, LV = left ventricle, IVC = inferior vena cava, PA = pulmonary artery, RL = right lung, PTV = planning target volume.

	**Contracted contours** **Alpha/beta of 2 Gy**	**Original contours** **Alpha/beta of 2 Gy**	**Expanded contours** **Alpha/beta of 2 Gy**	**Alpha/beta of 3 Gy** **Original contours**
**EN (Dose only)**	LA mean dose (87%) Other dose parameters (84%)	LA mean dose (71%) Other dose parameters (65%)	LA mean dose (91%) Other dose parameters (90%)	LA mean dose (66%) Other dose parameters (59–60%)
**EN (Complete)**	LA mean dose (74%) Other dose parameters (59–72%) Age (76%) Log(PTV) (87%) Pathology (77%)	LA mean dose (70%) Other dose parameters (60–66%) Age (74%) Log(PTV) (86%) Pathology (74%)	LA mean dose (77%) Other dose parameters (69–75%) Age (77%) Log(PTV) (88%) Pathology (78%)	LA mean dose (67%) Other dose parameters (56–65%) Age (71%) PTV volume (85%) Pathology (72%)
**RSF (Dose only)**	LV/IVC max dose, LA mean dose (0.02) LV mean dose (0.01)	LV max dose, LA mean dose (0.02) LA/IVC max dose, LV mean dose (0.01)	LA/LV/IVC max dose, LA mean dose (0.02) LV mean dose, RL max dose (0.01)	LV max dose, LA mean dose (0.02) LA/IVC/RL max dose, LV mean dose (0.01)
**RSF (Complete)**	LV/IVC/RL max dose, LA/WH mean dose (0.01) Age (0.02) Log(PTV) (0.03) Pathology (0.03)	LV/IVC/PA/RL max dose, LA/LV/WH mean dose (0.01) Age (0.02) Log(PTV) (0.03) Pathology (0.03)	LA/IVC/RL max dose, LV/WH mean dose (0.01) LA mean dose (0.02) Age (0.02) PTV (0.03) Pathology (0.03)	LA/LV/WH mean dose (0.02) LA/LV/IVC/PA/RL max dose (0.01) Age (0.02) PTV (0.03) Pathology (0.03)

In each configuration, LA mean dose remained the most selected dose parameter. PTV size was the most selected confounding factor (> 86%), while age (> 70%) and pathology (> 71%) were selected about as often as LA mean dose. All selection percentages are shown in Supplementary Materials 5.

The RSFs for the confounding factors mostly agreed with the ENs, showing PTV volume and pathology to be the most important, while also having age above the importance threshold. Interestingly, the difference in the model variations for the RSF was much more limited than that of the EN models. Several dose parameters met the importance threshold over the different model variations, but only the LA mean dose surpassed the threshold consistently. The relative importance metrics can be assessed and compared in the box plots in Supplementary Materials 6.

The LA mean dose was the most relevant dose parameter when combining the ENs and the RSFs results.

**Fig. 3:**
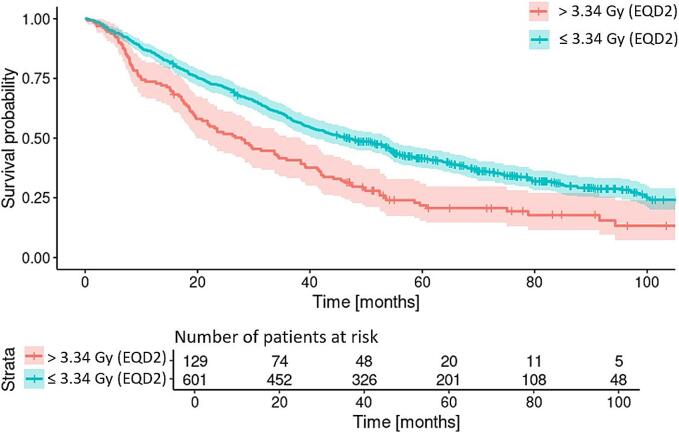
Kaplan-Meier plot using the identified LA mean dose threshold.

Using the LA mean dose for a new multivariable cox-regression model resulted in the following HRs [Confidence interval]: 1.02 [1.00–1.05] (original contours), 1.02 [1.00–1.05] (expanded contours), 1.02 [1.00–1.05] (contracted contours), and 1.02 [1.00–1.05] (α/β of 3 Gy). Outcomes of the full multivariable cox-regression model are shown in Supplementary Materials 4 Table 4. Next, the optimal split of the LA mean dose was determined to be 3.34 (original contours), 4.09 (expanded contours), 3.12 (contracted contours), and 3.64 (α/β of 3 Gy) Gy EQD2. The LA mean dose threshold was used to split the cohort and a Kaplan-Meier plot is shown in Fig. 3. Using this threshold as a stratifying factor led to the following difference in median survival: 46.2 vs 26.7 months for below vs above the thresholds respectively. Dose statistics on the CS for the contour variations and α/β of 3 Gy can be found in Supplementary Materials 1 Table 2, while clinically used dose constraints for conventional organs at risk are located in Supplementary Materials 1 Table 1.

## 4 Discussion

We have investigated the influence of CS dose on OS of lung cancer patients, after receiving SBRT, using EN and RSF models, while taking into account delineation-induced dose and α/β uncertainty, and the influence of confounding factors. The LA was the most critical CS, which is in agreement with findings by others [10], [15], [17], [35], [36]. The potential LA mean dose threshold should be validated in future studies. When consensus for CS constraints has been reached, they can be included in lung SBRT treatment planning as shown in a recent study [37]. The mean dose constraint found in this work suggests the LA acts as a parallel tissue. Atrial irradiation has been linked to irregular heart rhythm via a maximum dose parameter [35]. Alternatively, atria might act as parallel tissue where multiple damaged spots lead to re-entry arrhythmia. Additionally, irradiating large blood volumes, even at a low dose, might lead to lymphopenia and therefore worse survival [38], [39]. Setting ambitious requirements (median DSC *≥* 0.80 and MSD < 2 mm) for the auto-mated contour generation and using training data from the whole inclusion period, enabled sufficient auto-contouring performance in the entire population. Our method outperformed other auto-contouring methods and time-consuming manual delineations by experts based on the reported metrics [40], [41], [42]. For example, the median DSC over the four chambers was higher using our automated contours: 0.81 (Chin et al. [40]), 0.79 (Milo et al. [41]), and 0.90 (This study), also compared to manual contouring: 0.83 (Milo et al. 2021 [41]), 0.78–0.87 (Milo et al. 2020 [42]). A limitation was that our study only focuses on the large CS. Further improvements in contouring performance can potentially be made by enlarging the training dataset for the DL network. Increasing performance sufficiently to include smaller structures, like coronary arteries and valves, likely requires images with higher contrast, finer resolution, and reduced motion blurring, e.g., MRI or (gated) contrast CTs [25], [43]. Another future direction could be creating a DL network that can handle multi-modal images.

The RA was found to be crucial by McWilliam et al. [16], [44] using comparable methods of analysis. However, their studies differ from ours by the lower delivered dose per fraction, making direct comparison difficult due to uncertainties in the linear-quadratic model. Furthermore, they did not include the confounding factors in the models used for dose parameter selection. In our study, adding confounding factors showed a large effect on the EN selection percentages, but limited effect on the RSF outcomes. Both with and without confounding factors, LA mean dose remained the most prominent dose parameter. Furthermore, we showed that our results are robust to possible contouring inaccuracies by modelling these scenarios. The choice to use MSD *±* 2 SD for the expansion and contraction could be considered overly cautious. However, smaller values are expected to produce outcomes closer to the original contour set.

The main limitation of this retrospective study was missing data, as the cause of death, cardiac event and co-morbidity information are unknown. This information is essential in the creation of a better survival model for this patient population as also stressed by others [10], [16], [17], [45]. RAPID-RT is a recently started study collecting this data and adapting treatment where necessary [46]. Another limitation was the inclusion of only mean and maximum dose parameters. However, this is done to limit the already extensive multicollinearity problem, as reducing the number of correlated parameters is recommended [47]. Alternatively, the RSF could have been used to select parameters for the input of the EN model.

Modelled HRs can be difficult to translate to clinical practice. The determined optimal split is more interpretable and may therefore be preferred. However, one drawback of the optimal splits is that they cannot take into account the differences in the confounding factors between either group. The method of using two-third of the data as training should circumvent this as a single sample might be biased, where several bootstraps were used to reflect the entire dataset more realistically.

Our EN models and RSFs only include dose parameters resulting from one variation of contours, without combinations. This might resemble an unrealistic scenario, where there is either an accurate segmentation or systematic under/over-segmentation. However, randomly picking contour variations for each patient could lead to 3^730^ different contour combinations, which makes it unfeasible to explore all of them. Simpler approaches, like selecting one contour variation per structure for the model, quickly become unfeasible as there are 3^8^ (equalling 6561) possible combinations.

In conclusion, the left atrium was found to be the most influential CS from the four chambers and great vessels when looking at the influence of CS dose on OS in lung cancer patients. We propose further investigation into the left atrium for patients receiving SBRT (*≥*5 Gy per fraction): left atrium Dmean *≤* 3.3 Gy EQD2.

## CRediT authorship contribution statement

**Luuk H.G. van der Pol:** Methodology, Software, Formal analysis, Writing – original draft. **Jacquelien Pomp:** Data curation, Writing – review & editing. **Firdaus A.A. Mohamed Hoesein:** Data curation. **Bas W. Raaymakers:** Supervision, Writing – review & editing. **Joost J.C. Verhoeff:** Supervision, Writing – review & editing. **Martin F. Fast:** Conceptualization, Methodology, Writing – review & editing, Supervision.

## Funding

None.

## Declaration of competing interest

The authors declare the following financial interests/personal relationships which may be considered as potential competing interests: Bas W. Raaymakers: Research support from Elekta on workflow optimization for adaptive radiotherapy. Presentation for Elekta user meeting in San Antonio, 2022 on MRI guided radiotherapy. Travel support for attending and presenting at MEFOMP in Oman, 2023, on development of MRLinac. Elekta advisory board on adaptive treatment planning; International advisory board of Physics in Medicine and Biology. The department of Radiotherapy where B.W. Raaymakers is working receives hardware from Elekta for research on development of adaptive radiotherapy. Martin F. Fast: NWO (Dutch Research Council), Research Grant 17515 (payment to institution); NWO (Dutch Research Council), Research Grant 19484 (payment to institution); European Union H2020, Research Grant 945119 (payment to institution); AAPM/EFOMP TG-391, Lead of task group on 4D-MRI (unpaid). The other authors have no conflicts of interest.
