# Supplementary material for: The influence of cardiac substructure dose on survival in a large lung cancer stereotactic radiotherapy cohort using a robust personalized contour analysis
Source: Phys Imaging Radiat Oncol. 2024 Dec 1;32:100686. doi: 10.1016/j.phro.2024.100686 (PMC11663986; doi:10.1016/j.phro.2024.100686)
Supplement: Supplementary Data 1 [file mmc1.pdf]

1. Supplementary Material

Supplementary Material 1: Conventional OAR dose constraints and population dose statistics

Table 1: Displaying the current clinical dose constraints used for treatment planning. Dose values are in Gy EQD2.

Structure	dose metric	EQD2 optimal constraint [Gy]	EQD2 mandatory constraint [Gy]
Aorta	D0.5cc	99.5	165
Aorta	D5cc	43	70
Spinal cord	D0.1cc	50.5	50.5
Esophagus	D0.5cc	64	70
Esophagus	D5cc	45.5	45.5
Trachea	D0.5cc	76	82.5
Bronchus (left/right)	D0.5cc	76	82.5
Heart	D0.5cc	99.5	142.5
Heart	D15cc	58.5	58.5
Plexus	D0.1cc	58.5	58.5
Skin	D0.5cc	99.5	99.5
Skin	D10cc	82.5	82.5

Table 2: Displaying the dose statistics over the population. Dose values are in Gy EQD2. Arteries and lungs have no separate values in the last column, as α/β of 3 Gy was the default. WH and lungs have no expanded/contracted contours.

**Dose parameter**	**Median dose [range] (original)**	**Median dose [range] (expanded)**	**Median dose [range] (contracted)**	**Median dose [range] (α/β of 3 Gy)**
LA max	2.23 [0 - 197.47]	5.15 [0.01 - 257.69]	1.33 [0 – 198.99]	2.42 [0.01 - 172.36]
RA max	0.56 [0 - 209.14]	1.18 [0 – 261.36]	0.47 [0 - 166.07]	0.65 [0 - 182.15]
RV max	0.36 [0 - 60.63]	0.55 [0 - 95.56]	0.31 [0 - 59.29]	0.43 [0 – 54.54]
LV max	0.44 [0 - 168.94]	0.63 [0 - 277.56]	0.40 [0 - 150.59]	0.52 [0 - 148.34]
Aorta max	15.83 [0.17 - 212.10]	21.17 [0.02 - 285.65]	13.87 [0.02 – 184.25]	-
SVC max	6.74 [0.01 - 233.83]	9.40 [0.01 - 289.57]	6.06 [0.01 - 200.52]	-
IVC max	0.10 [0 - 141.82]	0.14 [0 – 220.58]	0.10 [0 – 112.43]	-
PA max	9.51 [0.02 - 264.54]	20.19 [0.08 – 363.12]	7.23 [0.01 – 239.44]	-
WH max	2.83 [0 – 229.98]	-	-	3.07 [0 – 199.61]
LA mean	0.35 [0 - 37.37]	0.43 [0 – 42.91]	0.31 [0 - 38.36]	0.39 [0 - 35.35]
RA mean	0.13 [0 – 47.31]	0.18 [0 – 43.75]	0.14 [0 – 49.60]	0.16 [0 – 44.80]
RV mean	0.08 [0 – 19.47]	0.12 [0 – 21.64]	0.09 [0 – 18.47]	0.10 [0 – 18.02]
LV mean	0.09 [0 – 24.15]	0.12 [0 – 23.37]	0.10 [0 – 23.72]	0.11 [0 – 23.64]
Aorta mean	1.67 [0.03 – 31.20]	1.87 [0 – 67.86]	1.78 [0 – 79.59]	-
SVC mean	1.65 [0 – 56.79]	4.76 [0 – 85.15]	1.60 [0 – 98.02]	-
IVC mean	0.06 [0 – 55.35]	1.03 [0 – 54.24]	0.06 [0 – 56.83]	-
PA mean	1.24 [0 – 96.46]	1.68 [0 – 83.22]	1.12 [0 – 103.25]	-
WH mean	0.25 [0 – 20.99]	-	-	0.29 [0 – 20.28]
LL mean	1.35 [0.02 – 50.47]	-	-	-
LL max	24.35 [0.11 – 594.51]	-	-	-
RL mean	1.96 [0.01 - 43.73]	-	-	-
RL max	220.54 [0.15 - 464.28]	-	-	-

Supplementary Material 2: Additional explanation on the used survival models.

Elastic net (EN) models in this work were used as a tool to determine which dose parameters are useful when predicting the survival time of a given patient. Conventionally cox-regression models are used for this, however, those models are not robust in handling multiple correlated parameters as input (known as the multicollinearity problem). An example we encountered was when first testing cox-regression models in this dataset was a positive influence on survival for higher doses to the aorta, which is highly implausible. In an attempt to resolve the multicollinearity problem, we chose to employ the EN models as was previously done by others in a similar setting [16] and is proven to be more effective compared to individual LASSO or Ridge modelling [48].

Our approach in using the EN models is having 1000 bootstraps, as single runs can vary from one another due to multiple parameters tuning. We ran 1000 bootstraps for each variation of the model. This means that each contour variation (original/contracted/expanded) as well as the test for an α/β value of 3 Gy, had one run with 1000 bootstraps. Additionally, this was done with and without the inclusion of confounding factors, thus creating eight variations in total. The results of these different variations were compared to one another for stability of the dose parameters. Additionally, these results were also compared to the RSF results (using the same variations) to investigate which dose parameters are useful regarding both methods.

The **random survival forests** (RSFs) in this work were used as a tool to determine which dose parameters are useful when predicting the survival time of a given patient. The survival time of the input population is modelled based on the parameters it is given. Identifying the importance of parameters is done by using permutations. During a permutation, the parameters of subjects are used to predict their survival, however, one parameter is permuted (a random sample of this parameter is drawn from the dataset). The predicted survival time from the model should now be different from the actual survival time per subject. If this is the case, the parameter is truly predictive of survival. When the predicted survival time stays the same or differs only slightly, the parameter that is being permuted is not/less predictive of the survival time. Doing this iterative over the complete set of parameters allows the model to determine the importance of each parameter. In this way, it can be determined if it is useful to include certain parameters in a survival model. The importance of the parameters is displayed in the results and Supplementary Materials. The RSF models were used in eight different variations (equal number to the EN models), three contour variations (original/contracted/expanded) and once using a different α/β value (3 Gy). These four variations were run with and without including confounding factors.

Supplementary Material 3: Auto-contouring test performance

Table 3: Results from auto-contouring of the n=10 test cases for all included cardio-pulmonary structures. Bold indicates structures used in the further analysis. Abbreviations: DSC - dice score, MSD - mean surface distance, SD - standard deviation

**Structure**	**DSC median (min - max)**	**95% HD [mm] median (min - max)**	**MSD [mm] mean (SD)**
**Left atrium**	**0.89** (0.86 - 0.91)	4.3 (3.1 - 6.0)	**1.3** (1.6)
**Right atrium**	**0.90** (0.80 - 0.91)	3.8 (3.0 - 8.0)	**1.4** (1.7)
**Right ventricle**	**0.87** (0.81 - 0.90)	6.0 (3.4 - 9.8)	**1.8** (2.1)
**Left ventricle**	**0.93** (0.90 - 0.95)	3.8 (3.1 - 7.2)	**1.2** (1.5)
Ascending aorta	**0.80** (0.00 - 0.84)	6.9 (3.0 - 24.5)	3.6 (3.3)
**Aorta**	**0.90** (0.85 - 0.95)	6.5 (2.7 - 18.0)	**1.3** (2.7)
Aortic valve	0.57 (0.23 - 0.69)	8.2 (6.0 - 11.0)	2.6 (2.8)
Coronary sinus	0.59 (0.23 - 0.71)	4.9 (3.3 - 12.2)	**1.7** (1.8)
**Superior vena cava**	**0.86** (0.75 - 0.91)	3.1 (1.8 - 23.4)	**1.1** (1.8)
**Inferior vena cava**	**0.81** (0.61 - 0.87)	5.1 (3.0 - 9.2)	**1.5** (1.9)
**Pulmonary artery**	**0.84** (0.80 - 0.90)	6.0 (3.3 - 22.7)	**1.6** (3.0)
Pulmonary veins	0.71 (0.57 - 0.78)	7.9 (3.2 - 15.4)	**1.8** (2.9)

Supplementary Material 4: Cox-regression analyses results

Table 4: Displaying the results of the univariable and multivariable cox-regression analyses. Abbreviations: HR = hazard ratio, CI = confidence interval, LA = left atrium, and CC = cranio-caudal.

**Variable**	**Univariable HR [CI]**	**Univariable p-value**	**Multivariable HR [CI]**	**Multivariable p-value**
Sex				
Male	Reference	Reference	Reference	Reference
Female	0.81 [0.68 – 0.97]	0.020	0.90 [0.74 - 1.08]	0.241
Tumour stage				
1	Reference	Reference	Reference	Reference
2	1.20 [0.98 - 1.46]	0.077	-	-
Pathology				
Neoplasm	Reference	Reference	Reference	Reference
Squamous	2.11 [1.59 - 2.81]	2.11 * 10^-7	1.76 [1.31 - 2.37]	1.7 * 10^-4
Adeno	1.38 [1.06 - 1.80]	0.018	1.30 [0.99 - 1.70]	0.054
Unknown	1.36 [0.96 – 1.94]	0.086	1.43 [0.99 - 2.06]	0.055
log(PTV volume)	1.33 [1.21 - 1.45]	5.79 * 10^-10	1.17 [1.05 - 1.31]	0.006
Age	1.02 [1.01 - 1.03]	3.93 * 10^-5	1.02 [1.01 - 1.03]	0.001
Laterality				
Left	Reference	Reference	Reference	Reference
Right	1.03 [0.86 - 1.22]	0.751	-	-
LA mean dose	1.05 [1.03 - 1.07]	5.44 * 10^-6	1.02 [1.00 - 1.05]	0.044
Distance to the heart				
PTV CC	1.00 [0.99 - 1.00]	0.053	1.00 [1.00 – 1.00]	0.464
PTV in-plane	1.00 [0.99 - 1.00]	0.286	-	-
GTV CC	1.00 [0.99 - 1.00]	0.342	-	-
GTV in-plane	1.00 [0.99 - 1.00]	0.090	-	-

Supplementary Material 5: EN selection percentages overview

Table 5: EN selection percentage overview for the model variations including only dose parameters. Abbreviations: LA = left atrium, RA = right atrium, RV = right ventricle, LV = left ventricle, SVC = superior vena cava, IVC = inferior vena cava, PA = pulmonary artery, WH = whole heart, LL = left lung, and RL = right lung.

**Model variation/ dose parameter**	**Contracted contours**	**Original contours**	**Expanded contours**	**Alpha/Beta of 3 Gy**
**LA max**	84.4	65.3	90.1	59.9
**RA max**	83.6	65.1	90.0	58.7
**RV max**	84.0	65.2	90.1	59.0
**LV max**	83.9	65.2	90.0	59.0
**Aorta max**	83.6	65.1	90.0	58.7
**SVC max**	83.8	65.2	90.0	58.8
**IVC max**	83.8	65.2	90.0	58.7
**PA max**	84.4	65.2	90.1	59.6
**WH max**	83.9	65.2	90.0	59.0
**LA mean**	**86.6**	**70.5**	**90.5**	**66.1**
**RA mean**	84.4	65.2	90.1	59.6
**RV mean**	84.4	65.2	90.1	59.8
**LV mean**	84.3	65.2	90.1	59.6
**Aorta mean**	83.6	65.1	90.0	58.7
**SVC mean**	83.7	65.1	90.0	58.7
**IVC mean**	83.6	65.1	90.0	58.7
**PA mean**	84.1	65.2	90.1	59.0
**WH mean**	84.4	65.3	90.1	59.9
**LL mean**	83.8	65.1	90.0	58.7
**LL max**	83.6	65.1	90.0	58.7
**RL mean**	83.9	65.2	90.1	59.0
**RL max**	83.6	65.1	90.0	58.7

Table 6: EN selection percentage overview for the model variations including dose parameters and confounding factors. Abbreviations: LA = left atrium, RA = right atrium, RV = right ventricle, LV = left ventricle, SVC = superior vena cava, IVC = inferior vena cava, PA = pulmonary artery, WH = whole heart, LL = left lung, RL = right lung.

Model variation/ dose parameter	Contracted contours	Original contours	Expanded contours	Alpha/Beta of 3 Gy
LA max	70.6	66.3	74.9	64.4
RA max	59.1	60.0	68.6	56.2
RV max	67.3	61.8	69.7	58.3
LV max	66.5	61.5	69.4	58.2
Aorta max	59.1	60.0	68.7	56.2
SVC max	63.0	60.7	69.3	57.2
IVC max	63.9	60.6	69.3	57.2
PA max	71.3	65.1	69.5	62.4
WH max	63.6	60.5	69.2	57.2
LA mean	**73.9**	**70.0**	**76.5**	**67.3**
RA mean	70.5	64.5	71.4	62.2
RV mean	71.8	65.9	72.9	64.7
LV mean	70.6	64.2	71.4	62.4
Aorta mean	60.6	60.0	68.6	56.3
SVC mean	59.0	60.0	68.6	56.2
IVC mean	59.1	60.0	68.7	56.2
PA mean	68.0	62.7	70.7	59.2
WH mean	71.8	66.2	72.3	63.8
LL mean	61.5	60.1	68.8	56.7
LL max	60.1	60.0	68.6	56.2
RL mean	65.9	61.4	69.5	57.9
RL max	59.0	60.0	68.6	56.2
Sex	68.1	62.5	70.1	59.1
Age	76.4	73.5	77.4	71.4
log(PTV)	87.2	86.3	87.6	85.1
Pathology	77.1	74.2	77.6	72.3

Supplementary Material 6: RSF complete importance boxplots.

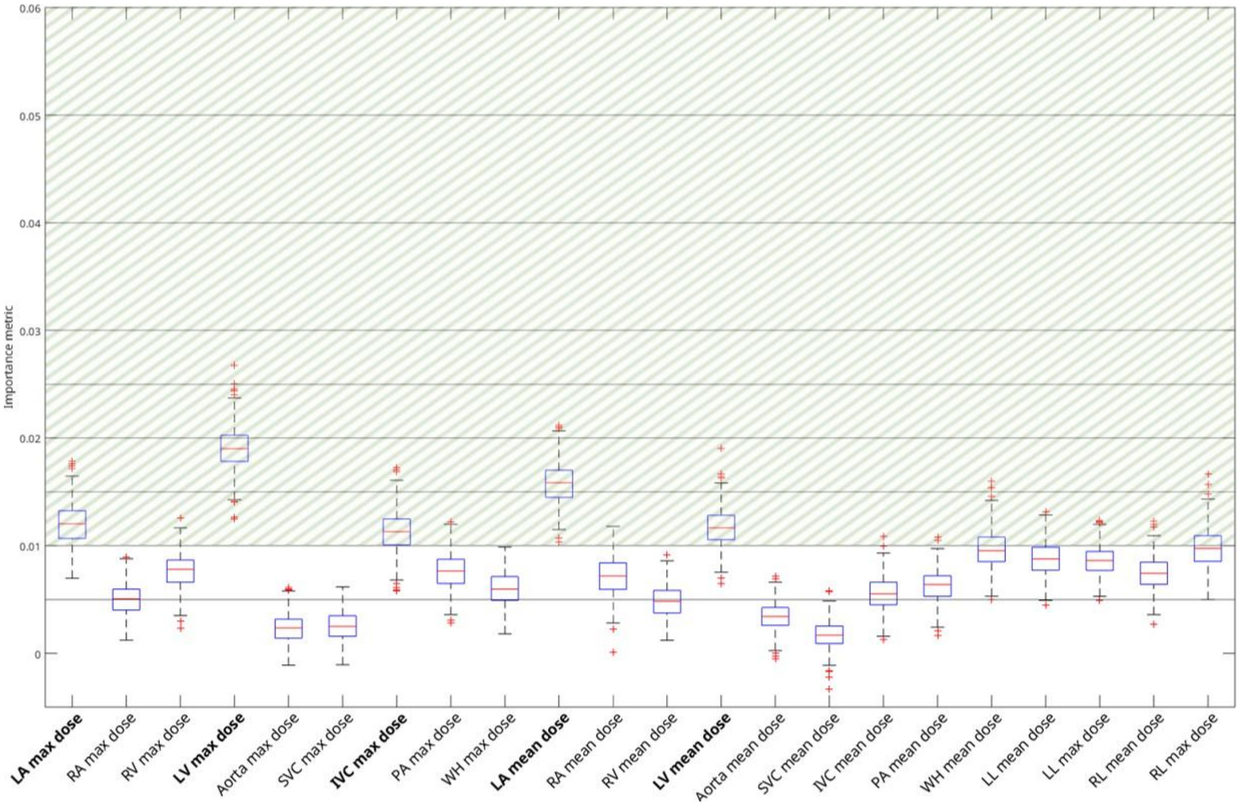

Figure 1: RSF using default contours, $\frac{\alpha}{\beta}$ of 2 Gy, and only dose parameters.

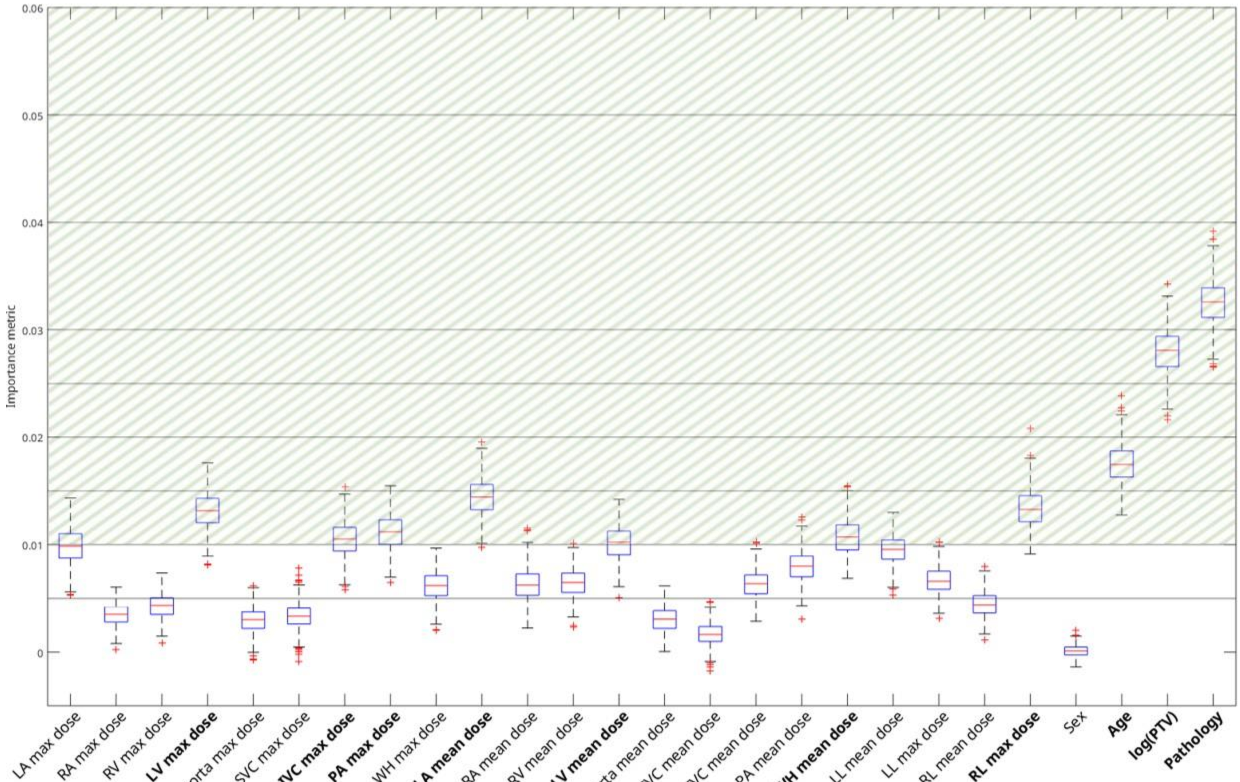

Figure 2: RSF using default contours, α/β of 2 Gy, and all parameters.

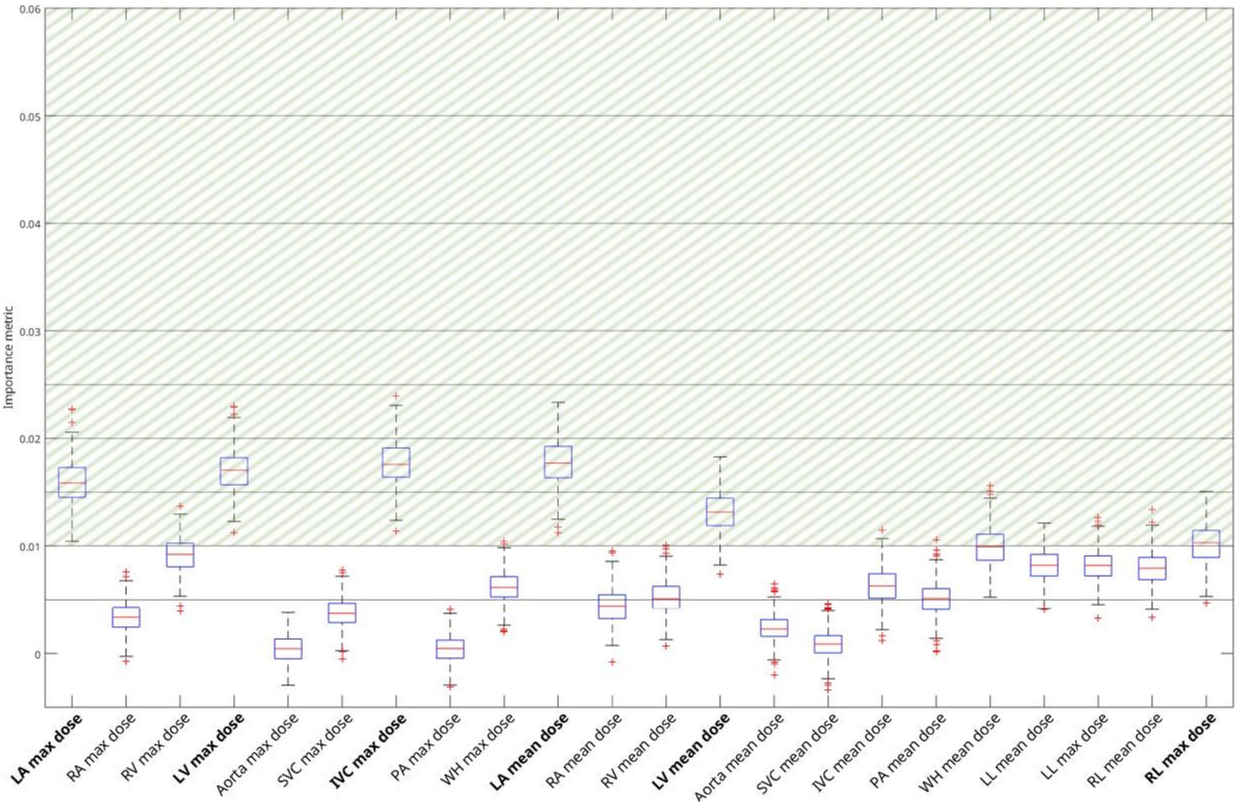

Figure 3: RSF using expanded contours, $\frac{\alpha}{\beta}$ of 2 Gy, and only dose parameters.

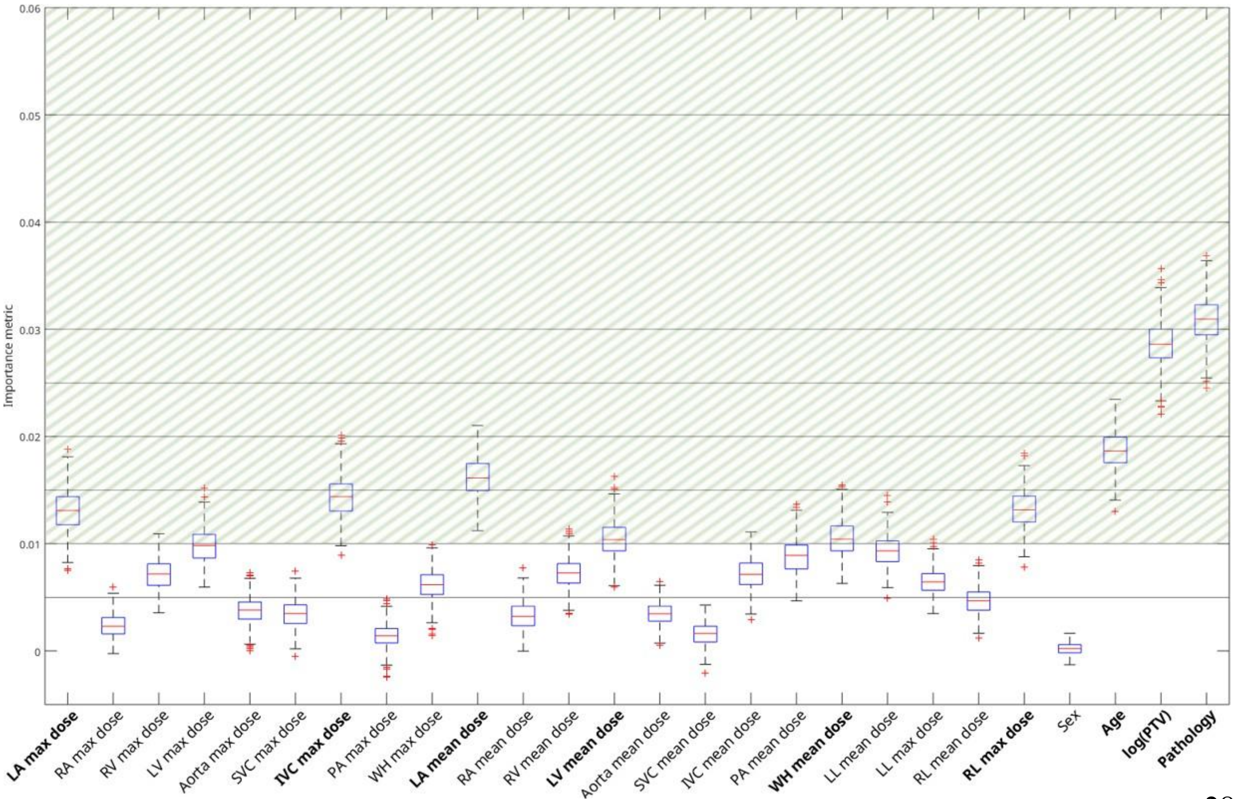

Figure 4: RSF using expanded contours, α/β of 2 Gy, and all parameters.

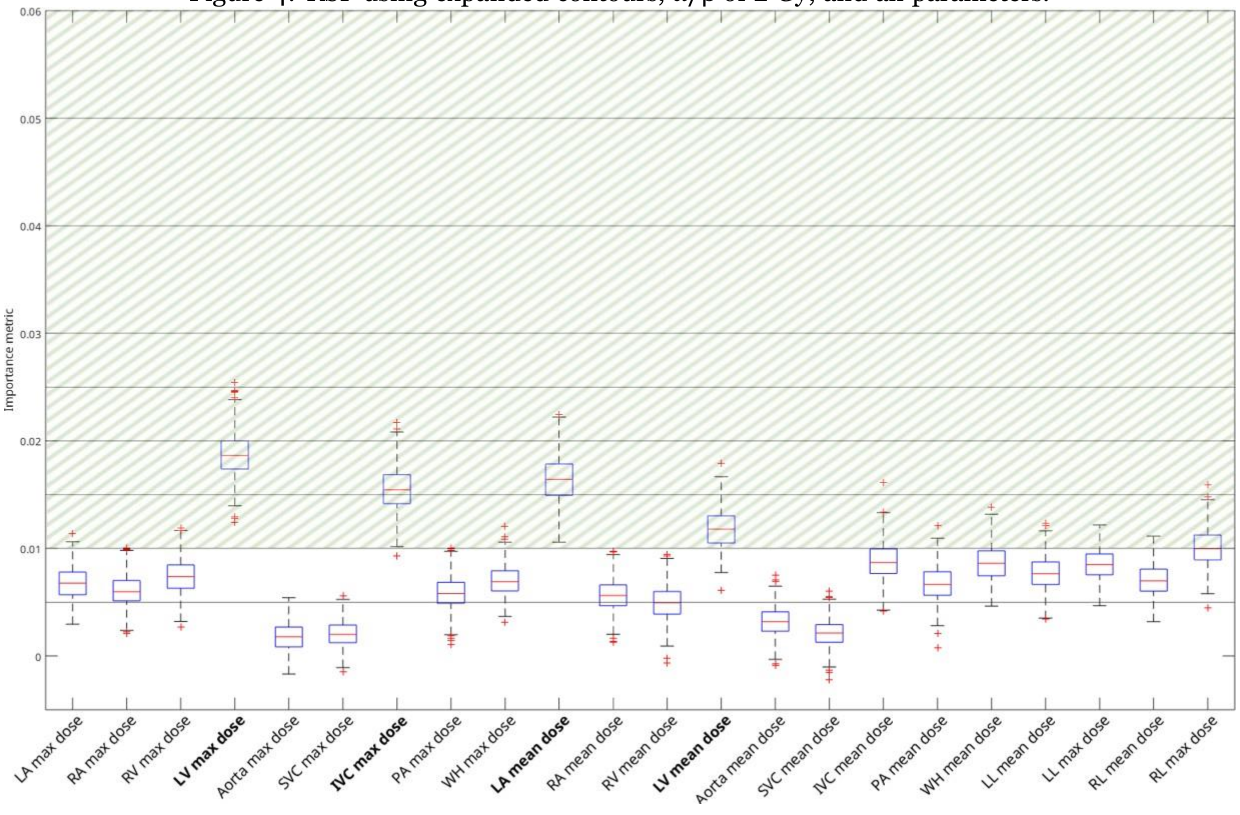

Figure 5: RSF using contracted contours, α/β of 2 Gy, and only dose parameters.

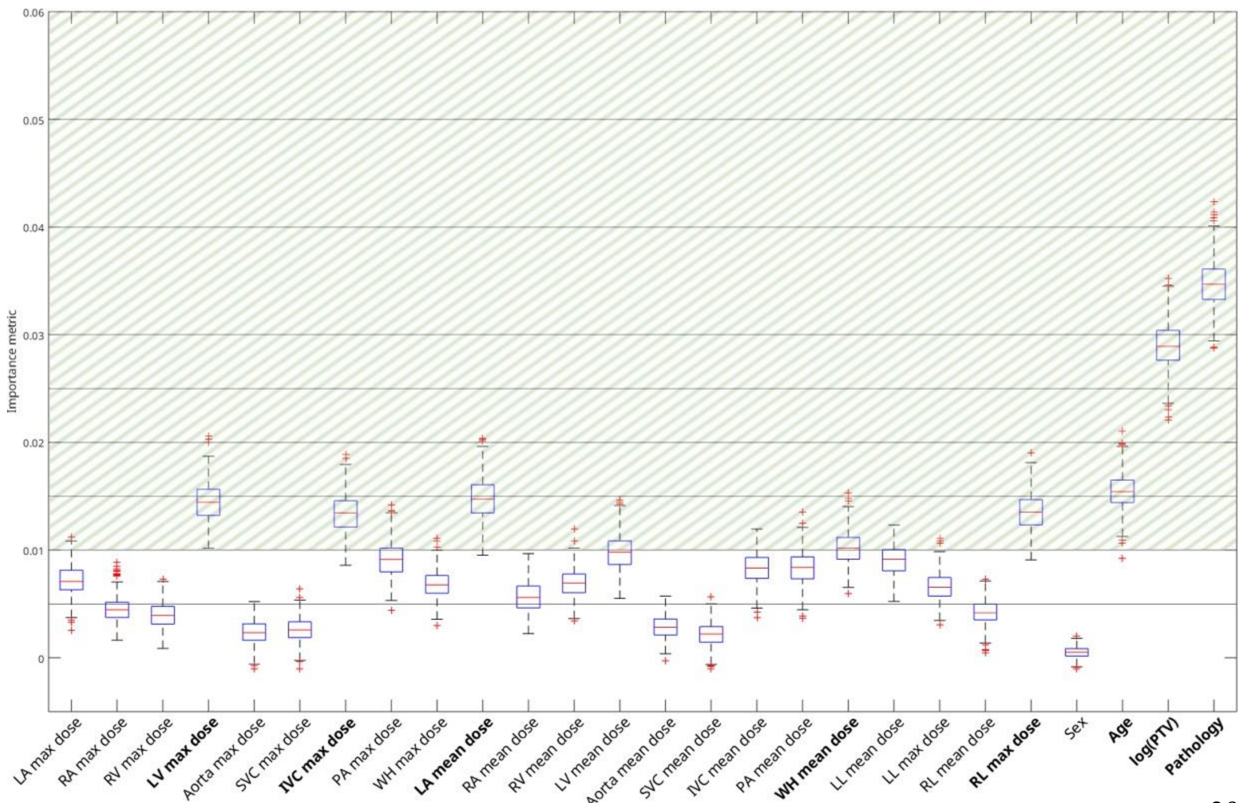

Figure 6: RSF using contracted contours, α/β of 2 Gy, and all parameters

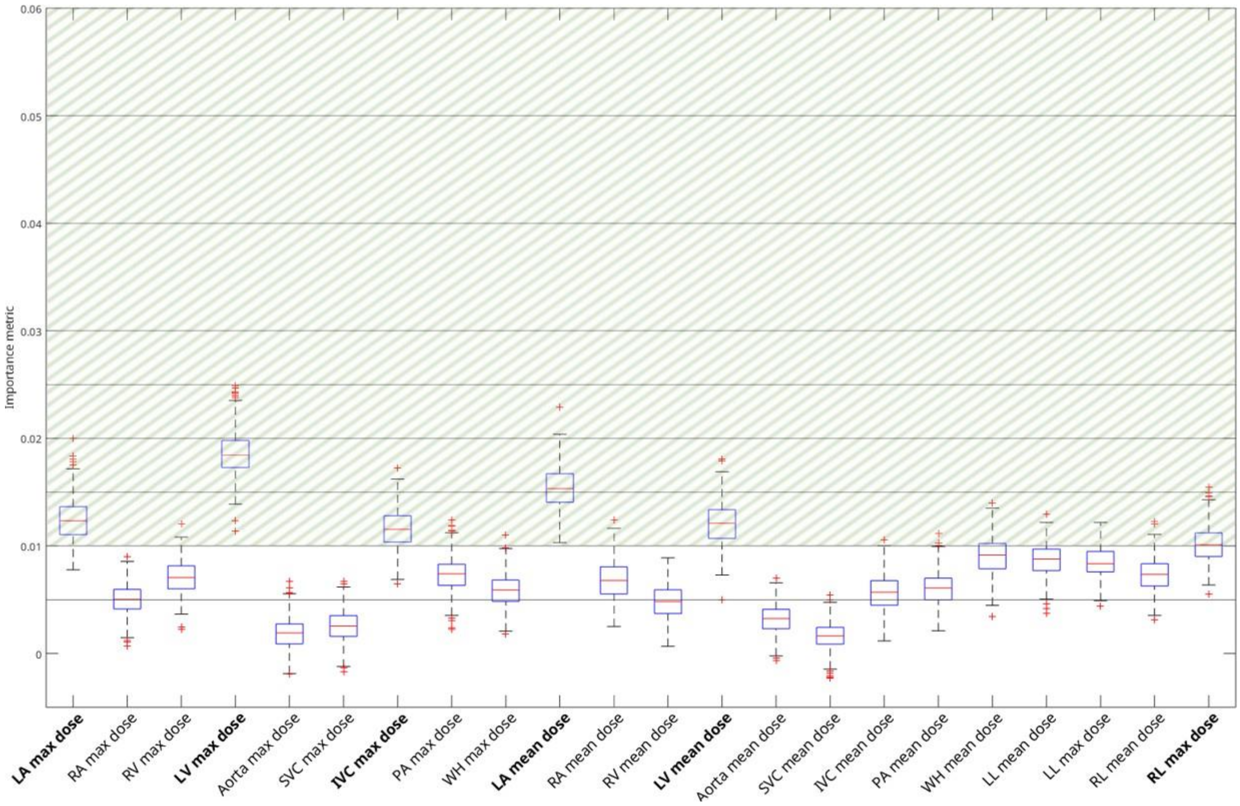

Figure 7: RSF using original contours, α/β of 3 Gy, and only dose parameters.

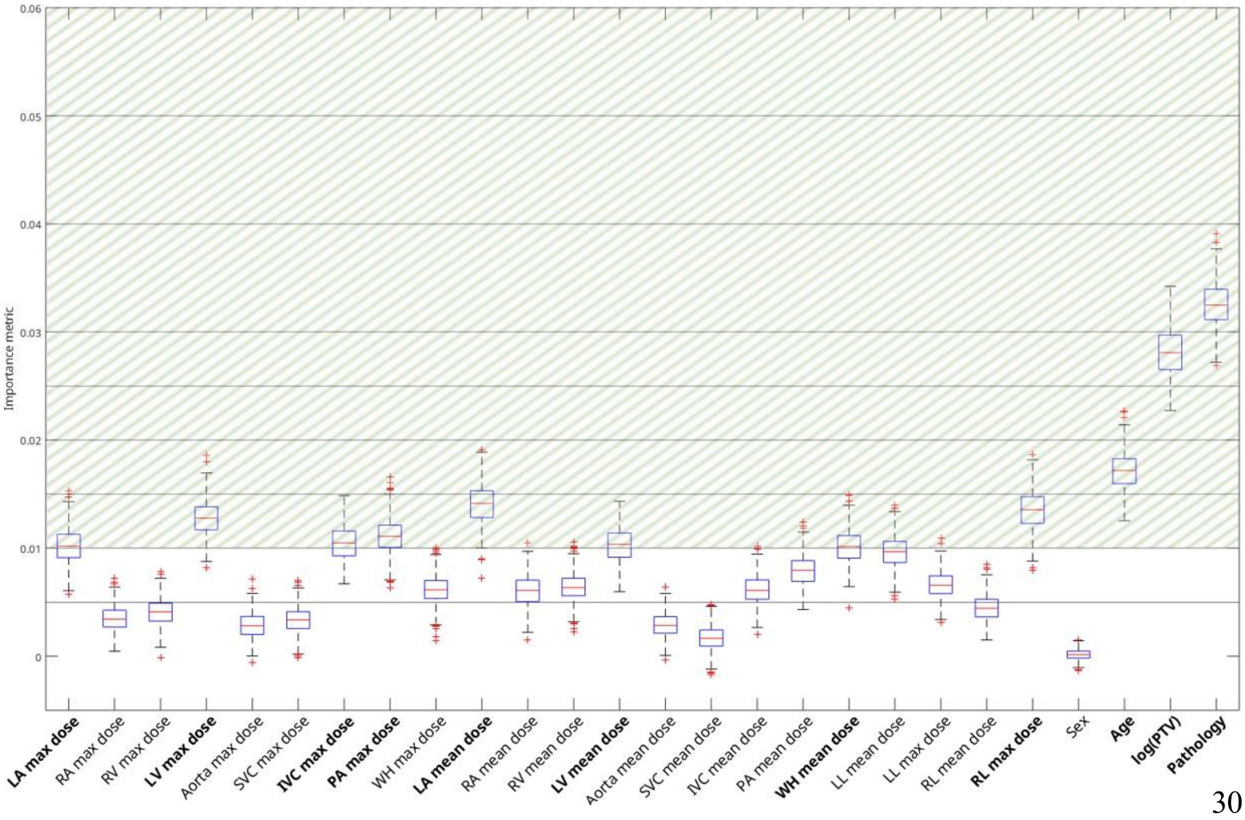

Figure 8: RSF using original contours, α/β of 3 Gy, and all parameters.

Supplementary References:

[16] McWilliam A, Khalifa J, Vasquez Osorio E, Banfill K, Abravan A, Faivre-Finn C, et al. Novel methodology to investigate the effect of radiation dose to heart substructures on overall survival. Int J Radiat Oncol Biol Phys 2020;108;1073–1081. doi: 10.1016/j.ijrobp.2020.06.031

[48] Herawati N, Wijayanti A, Sutrisno A, Nusyirwan, Misgiyati. The performance of ridge regression, lasso, and elastic-net in controlling multicollinearity: A simulation and application. J. Mod. Appl. Stat. Methods 2024;23. doi: 10.56801/Jmasm.V23.i2.3.
